# Pigs in Space: Determining the Environmental Justice Landscape of Swine Concentrated Animal Feeding Operations (CAFOs) in Iowa

**DOI:** 10.3390/ijerph13090849

**Published:** 2016-08-25

**Authors:** Margaret Carrel, Sean G. Young, Eric Tate

**Affiliations:** 1 Department of Geographical & Sustainability Sciences, University of Iowa, Iowa, IA 52242, USA; sean-young@uiowa.edu (S.G.Y.); eric-tate@uiowa.edu (E.T.); 2 Department of Epidemiology, University of Iowa, Iowa, IA 52242, USA

**Keywords:** environmental justice, swine, concentrated animal feeding operation (CAFO), Iowa

## Abstract

Given the primacy of Iowa in pork production for the U.S. and global markets, we sought to understand if the same relationship with traditional environmental justice (EJ) variables such as low income and minority populations observed in other concentrated animal feeding operation (CAFO) studies exists in the relationship with swine CAFO densities in Iowa. We examined the potential for spatial clustering of swine CAFOs in certain parts of the state and used spatial regression techniques to determine the relationships of high swine concentrations to these EJ variables. We found that while swine CAFOs do cluster in certain regions and watersheds of Iowa, these high densities of swine are not associated with traditional EJ populations of low income and minority race/ethnicity. Instead, the potential for environmental injustice in the negative impacts of intensive swine production require a more complex appraisal. The clustering of swine production in watersheds, the presence of antibiotics used in swine production in public waterways, the clustering of manure spills, and other findings suggest that a more literal and figurative “downstream” approach is necessary. We document the presence and location of antibiotics used in animal production in the public waterways of the state. At the same time, we suggest a more “upstream” understanding of the structural, political and economic factors that create an environmentally unjust landscape of swine production in Iowa and the Upper Midwest is also crucial. Finally, we highlight the important role of publicly accessible and high quality data in the analysis of these upstream and downstream EJ questions.

## 1. Introduction

**Figure 1:**
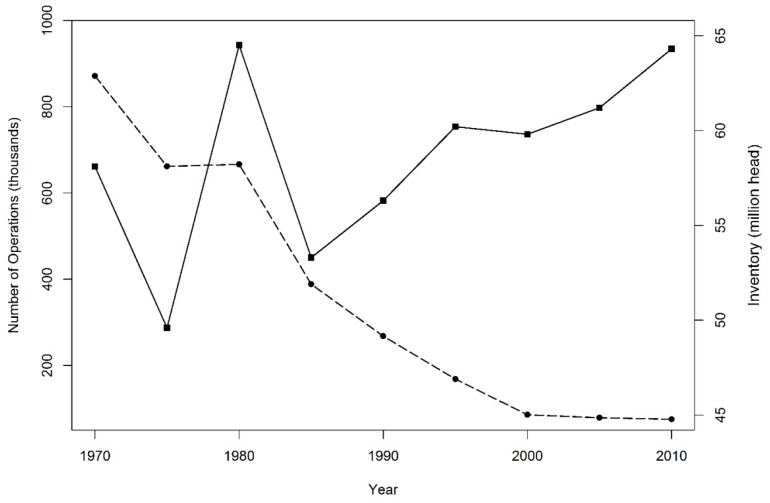
Number of swine operations in the U.S. over time (dashed line) versus the inventory of swine during that same time (solid line) [5].

Global demand for meat is increasing as the emerging middle class in low and middle-income countries undergoes a nutrition transition [1,2,3,4]. The response to this increased demand for meats such as pork, beef and poultry is a phenomenal escalation of livestock production. The United States (U.S.) is second only to China in the tonnage of pork produced annually, and the U.S. is responsible for nearly a third of global pork exports. Although pork production in the U.S. has risen in the previous decades, the number of sites where swine are raised has decreased. Pork production in the U.S. is highly concentrated in a relatively small number of farms even as the number of swine grown in those farms has increased (Figure 1). Individual swine farms themselves might have quite large geographic footprints, with multiple barns and large lagoons for the disposal of manure and other effluent.

This concentration of animal production into relatively fewer numbers of operations, termed concentrated animal feeding operations (CAFOs), has generated concerns about the concentration of negative externalities associated with intensive livestock production [6]. Chief among these are air and water pollution, and their effects on human health [7,8]. The potential for serious and harmful impacts of CAFOs on water quality results in their primary regulation taking the form of Clean Water Act standards as implemented through state environmental agencies [9]. CAFO operators must ensure that the byproducts of livestock production, namely manure and all its contents (urine, feces, viruses, bacteria, chemicals, antibiotics, etc.), are properly disposed of and do not threaten ground or drinking water.

The primary location of swine production in the U.S. is the Upper Midwest, particularly Iowa. In 2014, North Carolina produced 13% of the swine in the U.S., while Iowa produced 31.4% (followed by Minnesota (12%), Illinois (6.9%) and Indiana (5.5%)) [10]. Nearly one third of all swine raised in the US live in Iowa, and over half live in four contiguous Midwestern states (for historical context of the role of Iowa and the Midwest in swine production, see [11]).

Several factors contribute to this continued geographic focus of swine production in Iowa and the Upper Midwest. Under current manure disposal regulations, a large area of farmland is required to support the safe disposal of the huge amounts of manure produced in swine production. Iowa has a larger acreage of appropriate farmland than do other states and can thus support a larger number of swine. Additionally, swine in U.S. CAFOs are fed a diet mainly of corn and soy, crops grown primarily in Iowa and the Upper Midwest; locating swine production in Iowa and other Midwestern states reduces the transport costs of these feedstuffs. Furthermore, Iowa has a regulatory environment that is friendly to large agribusiness, making the production and slaughter of swine in the state an attractive option for major pork corporations, such as Hormel, Smithfield and Cargill. Iowa was investigated by the Environmental Protection Agency in 2012 for failing to regulate CAFOs as required under the Clean Water Act. Additionally, Iowa has passed laws that prohibit the state from regulating CAFOs more than required by federal law, and the Iowa Supreme Court ruled that local (county) governments cannot enforce stricter regulation than the state (removing local ability to fight CAFO locations) [12]. Environmental pollution from CAFOs, as described above, presents the potential for adverse human impacts, but these impacts may be differentially experienced by certain segments of the population.

Environmental justice (EJ) refers to the disproportionate burden of environmental hazards on marginalized populations, and the associated social activism of affected communities. EJ definitions vary widely, and include framings as distributive, procedural, corrective, social, and intergenerational justice [13]. Analysis of distributive justice focuses on inequities in the distribution of human exposure to environmental hazards, and has typically been explored quantitatively through statistical comparisons of environmental hazards and the demographic characteristics of nearby places. Studies using a procedural justice framing examine the political, economic, social, legal, technological, and institutional barriers and opportunities that shape levels of community involvement in environmental decision-making [14,15]. EJ research from a corrective justice perspective has examined the remedies, pace, and management of environmental remediation and their effects on impacted communities [16,17]. Social justice approaches situate environmental inequities and social activism within interrelated racial, economic, and political power structures that confer privilege to some and disadvantage to others [18,19]. Evaluation of intergenerational justice centers on long-term states of environmental equity and impact [20].

As a field of academic inquiry, the short history of EJ is characterized by steady expansion in the hazards investigated and the theory and methods applied [21,22,23]. Initial work focused on documenting the existence of environmental injustices associated with industrial and municipal hazards, primarily using statistical and spatial approaches to investigate the distribution of hazards and effects [24,25]. Over time, studies began to adopt more nuanced theoretical perspectives on inequality that move away from reflexive analysis of race and poverty variables, toward consideration of a wider array of demographic characteristics that reflect not only inequitable distributive outcomes, but also the underlying social, historical, political, and economic processes that produce them [26,27,28]. More recently, the domains of EJ assessment have expanded to include health, natural hazards, climate, resource extraction, transnational pollution, environmental amenities, and the focus of this study, agricultural production [29,30,31,32,33]. An overarching finding of EJ research is that racial minorities, ethnic minorities, indigenous populations, and low-income populations are disproportionately burdened with higher hazard incidence and exposure [34,35].

The bulk of quantitative EJ research regarding U.S. CAFO-related hazards has been situated in North Carolina. Collectively, the findings indicate an uneven distribution of swine CAFOs in the state, and thus potentially unevenly distributed negative externalities of swine production. Swine confinements were significantly more likely to be found in places with higher proportions of residents who were racial/ethnic minorities and living in poverty [36,37,38,39,40,41]. Similar relationships between uneven CAFO exposure and race and income were also observed in Mississippi [42]. The factors of race, ethnicity, and socioeconomic status are typically included in U.S. environmental justice quantitative analyses of exposure to environmental contaminants, ranging from hazardous waste to air pollution [43,44,45,46,47].

Despite the prominence of race, ethnicity, and class in previous studies, disparities in these factors are not universal prerequisites of environmental injustice. At its roots, environmental injustice stems from differentials in economic, social, and political power that lead to disproportionate hazard exposure. These differentials often manifest via race, ethnicity, and class, but may also be associated with other societal characteristics such as age and gender. Recent studies have considered if rural residents should also be included as members of EJ communities [48,49]. The relative influence of these and other causal variables is context specific, meaning their importance may vary from place to place.

This article describes a quantitative analysis of distributive justice associated with agricultural pollution from swine CAFOs. Given the primacy of Iowa in swine production for the U.S. and global markets, we sought to understand if the same relationship with traditional environmental justice variables such as low income and minority populations observed in other CAFO studies was found in relation to swine CAFO densities in Iowa. The clustering of swine production in watersheds, the presence of antibiotics used in swine production in public waterways, the clustering of manure spills and other issues investigated in the paper suggest that a more literal and figurative “downstream” approach is necessary. At the same time, a more “upstream” understanding of the structural, political and economic factors that create an environmentally unjust landscape of swine production in Iowa and the upper Midwest is also crucial.

## 2. Materials and Methods

Data on CAFOs were gathered from the Iowa Department of Natural Resources (DNR), which monitors CAFOs under Clean Water Act legislation. For each CAFO, the total number of swine animal units was calculated, and only CAFOs with swine units of greater than zero were retained for analysis. Animal units are used by the Iowa DNR to standardize the measure of manure and other waste generated by animals of varying sizes. A fully-grown cow equals a single animal unit, and market weight swine (hogs) equal approximately 0.4 animal units. Pigs (swine < 120 pounds) and chickens are even smaller fractions of an animal unit. Each CAFO was geocoded to a specific latitude and longitude by the DNR based on street addresses and photo interpolation, and these coordinates were utilized in a Geographic Information System (GIS) for spatial analysis.

**Figure 2:**
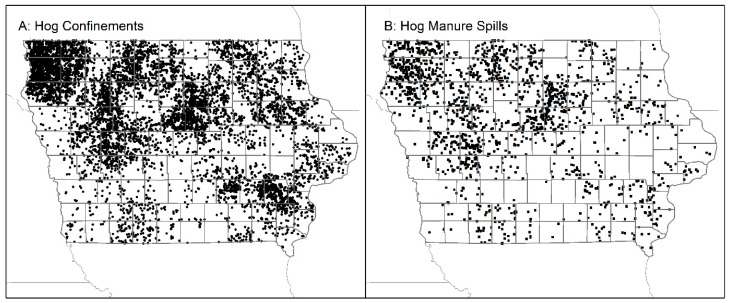
Locations of swine CAFOs (**A**) and swine manure spills (**B**) in Iowa.

Density measures of swine AUs were generated to account for highly varying sizes of units used in subsequent analysis. Data on the location and number of swine AUs in Iowa CAFOs were accessed from the DNR in September of 2015 (Figure 2). Only CAFOs designated as currently operating were retained for analysis. The total number of swine AUs in each facility was summed. To account for the potential impacts of swine confinements across administrative boundaries, kernel density estimation calculating a measure of swine AUs per square mile was generated in ArcMap (v. 10.3, Esri, Redlands, CA, USA). The mean density of swine AUs per square mile was then calculated for Census Block Groups (CBGs) and watersheds (Hydrological Unit Code (HUC)-8 scale). The locations of CAFOs were then aggregated by CBG and HUC-8 watershed, using boundaries made available by the U.S. Census and Iowa DNR. These sums of swine AUs were then standardized by the area of each CBG and watershed, generating an estimate of swine AU density per square mile.

By creating a raster surface of swine AU density across the state, rather than simply assigning CAFOs to CBGs or watersheds based on their precise location, two problems were addressed. The first problem is that the Iowa DNR assigns an area, the CAFO, to a point rather than a polygon of space and this point could be located outside of the CBG or watershed in which it actually exists because of imprecision or inaccuracy in the location taken by the Global Positioning System (GPS). The second issue is that a CBG may not contain any CAFO points but there could be multiple CAFOs located along that CBG’s border whose impacts affect populations in the so-called “zero swine AU” CBG. A “spatial coincidence” methodology, wherein CAFOs are assigned to the area in which they fall, would underestimate the impact of those bordering CAFOs given that the distribution of water and air pollution are not impacted by Census administrative boundaries [50,51]. At the same time, however, assigning an zone of influence around a CAFO, as is sometimes done when proximity to a hazard is the focus of analysis, is difficult when the true downstream or downwind or larger economic and ecological consequences of swine production is unknown. For these reasons, a density measure within CBGs and watersheds that includes the influence of CAFOs outside of the strict administrative or hydrologic bounds was used.

The spill of manure is a reportable event, and each manure spill reported to DNR is entered into a database of all hazardous releases. The DNR hazardous release database was accessed in January 2016. Spills are supposed to be reported within six hours of realization that an event has occurred. Each release is associated with a latitude and longitude coordinate, the type of substance spilled, and, infrequently, an estimate of the amount of substance spilled. The location of spills where swine manure is indicated, or manure is listed and the source is not poultry or cattle, were included for further analysis. The density of swine manure spills was calculated via the same kernel density estimation, as described above for swine AU density, taking into account the potential influence of a manure spill located across a CBG or watershed border.

From the five-year 2009–2013 American Community Survey (ACS), we generated traditional EJ measures at the scale of the CBG. The ACS replaces the long form questionnaire previously used in the decennial Census and is available at different time scales than the traditional Census (i.e., in one, three or five year sets rather than every ten years). The ACS is designed to be statistically representative of the U.S. population but is a survey rather than a complete census. The EJ measures chosen for analysis were the percentage of residents whose primary race/ethnicity was non-white, the percentage of residents with less than a college education, and the percentage of residents living in poverty. An additional measure of population density in CBGs was generated to explore the relationship between swine AU density and how the total population in Iowa is distributed across space. The percentage of residents in a CBG with less than a college education was chosen rather than the more typical less than high school measure because of the high rate of high school completion across Iowa. Charting the percentage of residents who do not complete high school versus those who do not attend or complete a college degree shows that most CBGs have very low percentages of residents who did not graduate from high school (no CBGs have percentages >60%) but that many CBGs have very low rates of college degrees (Supplemental Figure S1). The less than college variable captures not only residents who did not complete high school but also those who did not attend college following high school or attended but did not complete a bachelor or associate degree. For these reasons a less than college variable was included in subsequent analysis as a marker of potentially limited access to power or other resources.

Descriptive maps indicating the spatial distribution of swine CAFOs and manure spills were created in ArcMap. Maps of swine AU density and manure spill density generated via kernel density estimation were also created, as were descriptive maps of the EJ covariates of interest. Urban areas, as defined by the 2010 Census, are indicated on the EJ covariate maps.

To test for the presence of non-random spatial patterns in the location of swine CAFOs and high swine density in Iowa, we employed a global test for spatial autocorrelation (a Getis-Ord General G) and a local hotspot analysis via the Getis-Ord Gi* test [52]. The General G test examines high or low values are for an entire study area. In contrast, the Gi* test indicates where in the study area high or low values cluster spatially, and evaluates a null hypothesis that no spatial autocorrelation is present in the phenomena of interest, that like values are not observed among spatial neighbors. Neighbors were identified using queen contiguity such that all features sharing any portion of a border with the feature under consideration were considered neighbors. A contiguity rather than distance, definition of neighbors was used because of the areal nature of the units of study, CBGs, and to avoid making assumptions about how far the influences of high swine density extend across space. The weights for the neighbor matrix were row standardized such that the total weight for each observation (CBG) sums to one. Row standardization is recommended when an aggregation scheme, in this case the assignment of kernel density estimates of swine density from point CAFO features to CBGs, is imposed upon the data. The sum of values (swine AU density or manure spill density) for each feature and its spatial neighbors is compared to the sum of all features to estimate an expected value, with the Gi* statistic providing a z-score indicating the likelihood of observing the clustered values by chance. Features with Gi* scores over 1.96 (*p* < 0.05) and 2.58 (*p* < 0.01) were identified as hotspots. The density of swine animal units per square mile within CBGs and watersheds were evaluated for the presence of spatial hotspots, and hotspots of high values of swine density were mapped. The manure spill density per square mile in CBGs and HUC-8 watersheds was also assessed for hotspots.

To visualize the raw swine AU density and EJ variables, parallel coordinate plotting was used. Parallel coordinate plots chart the values associated with one observation (CBG) across multiple dimensions (swine density, EJ variables, population density) and are a useful tool for displaying patterns or trends in data, albeit without statistical testing or consideration of the spatial relationship among observations. To formally test the relationship between swine AU density and the demographic characteristics of Iowa CBGs, spatial regression was used.

In traditional linear, logistic or other forms of regression, an underlying assumption of the method is that observations, and their error terms, are independent of one another. When evaluating the arrangement of people or other phenomena in geographic space, however, this assumption of independent observations is rarely true. Regression methods that do not take into account the spatial relationship among observations, and the influence of neighboring observations on the outcomes of data points, overestimate the significance and effect of covariates on outcome variables, misattributing correlation to independent variables that is really a product of spatial autocorrelation. Research in environmental justice, as well as other fields, have indicated that significant relationships are observed between environmental hazards and EJ populations after spatial dependence among observations is taken into account, indicating environmental injustice is present despite spatial autocorrelation in human geography [33,47,53,54,55,56,57].

First, bivariate and multivariable Ordinary Least Squares (OLS) regressions with swine AU density as the outcome variable and EJ variables as the predictor variables were conducted and regression diagnostics, such as tests for spatial dependence, were examined. The potential presence of multicollinearity among EJ covariates was assessed via a multicollinearity condition number (where values > 30 indicate collinearity) and variance inflation factor (VIF) scores (where scores > 10 indicate collinearity) [58,59]. Spatial regression was then conducted. Spatial regression accounts for the spatial dependence among observations via the use of spatial weights; these weights indicate the location of neighbors with influence on the relationship between covariates and the outcome variable. A first order queen contiguity-weighting scheme with row standardization, as has been previously described, was used. A spatial lag model was used to account for the spatial dependence in the outcome variable, swine AU density. A second model that incorporated population density was also examined, to account for the widely varying population geography of rural versus urban Iowa. All OLS and spatial regressions were carried out in Open GeoDa (Center for Spatial Data Science, University of Chicago, Chicago, IL, USA), freely available software for spatial analysis. Open GeoDa does not calculate VIF scores; these were generated using the OLS functionality in ArcMap.

To explore the potential for the negative impacts of swine CAFOs on local water sources, we examined the presence of antibiotics used in animal production in water used by humans. Managed by the Watershed Monitoring and Assessment Section of the Iowa Geological and Water Survey Bureau, the STORET/WQX database stores results of water samples from rivers, lakes, groundwater and wetlands within the state of Iowa since 1999. The number and distribution of water quality monitoring sites is constantly changing. We used results from 81 continuously monitored sites, and measured the proportion of test results at each location with detectable levels of antibiotics used in swine production that also have relevance for human health. Seven analytes (lincomycin, sulfadimethoxine, sulfamethazine, sulfamethoxazole, sulfathiazole, trimethoprim, and tylosin) are utilized in swine production as well as treatment of bacterial infections in humans and were present in the STORET database [60,61,62,63,64,65,66]. Testing was performed to detect each analyte at each location, with the exception of tylosin, which was not present in the database after 2005. The median number of test samples per site was 18 (Interquartile Range (IQR) 17–20) for all analytes except tylosin, for which the median number of tests per site was 4 (IQR 1–9). Maps were generated to demonstrate the proportion of water samples that tested positive for each antibiotic. Analyte test results were further aggregated to the watershed scale and plotted against swine AU density and manure spill density in each watershed to assess potential correlation between swine production and antibiotic presence in public waterways.

## 3. Results

Data downloaded from the Iowa DNR indicate the presence in Iowa of 7105 CAFOs with swine AU counts of greater than 0 and a status of actively in operation (Figure 2). Dating the construction of CAFOs is difficult, as many are missing records for date of permitting, permits may be issued months before production begins, and a CAFO could get re-permitted that was already in existence. The 7105 CAFOs represent those that were in operation as of September 2015. A total of 2057 manure spills from 2005–2015 listed as either swine waste (1204, 59%) or where the animal source was blank (853, 41%) occurred were included in the dataset of hazardous spills. The amount of manure spilled was missing from 1572 (76%) of the records. The majority of swine CAFOs are located in the northwest, central and southeastern portions of the state, although no county is without a confinement. Spills of swine manure exhibit a similar, though less dense, pattern.

**Figure 3:**
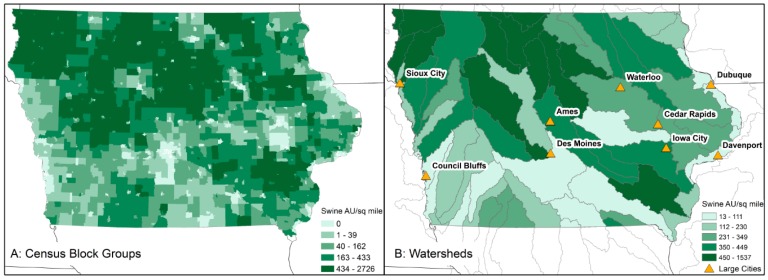
Swine AU density by CBG (**A**) and watershed (**B**) in Iowa. Larger cities (>50,000 people) are represented in (**B**).

Descriptive mapping of the swine AU density at the CBG and watershed scale suggested that a non-random and uneven distribution of swine might be present (Figure 3). Generally speaking, areas in the northwest, central and southeastern portions of Iowa have higher swine densities than the rest of the state, with the lowest densities of swine found in the areas surrounding Iowa’s cities, such as Des Moines, Waterloo and Iowa City. High swine AU density watersheds are, in some cases, those that drain into some of Iowa’s largest cities, such as Des Moines and Sioux City (Figure 3B).

**Figure 4:**
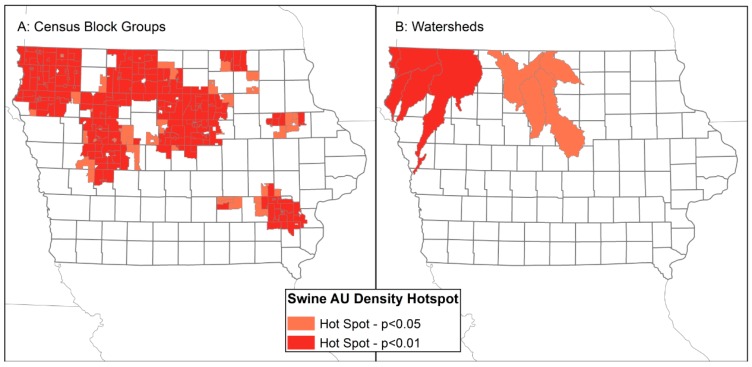
Hotspots of high swine animal unit density in Census block groups (**A**) and watersheds (**B**).

Significant global spatial autocorrelation exists for swine AU densities in both CBGs and watersheds in Iowa (CBG z-score = 46.8, *p* < 0.001; watershed z-score = 4.3, *p* < 0.001). Hotspot analysis shows many CBGs with high densities of swine AUs that are neighbored by other CBGs with high swine AU density (Figure 4A). These are located primarily in the northern portions of the state with a grouping of high swine density CBGs in the southeast. Approximately 11% of the CBGs in Iowa are located in a high swine AU density hotspot. Significant hotspots of high swine AU density are also observed in north-central watersheds (Figure 4B). Because the determination of a hotspot includes the densities in neighboring units, some CBGs and watersheds that themselves contain high swine AU densities but are surrounded by areas with relatively lower AU densities are not significant hotspots. Southeastern Iowa, for instance, has high swine AU densities in some watersheds but not in neighboring watersheds that constitute a hotspot.

**Figure 5:**
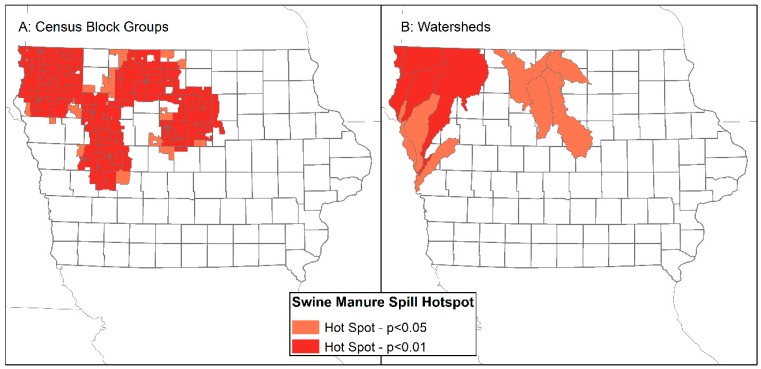
Hotspots of high swine manure spill density in Census block groups (**A**) and watersheds (**B**).

Within Iowa, manure spills also exhibit significantly non-random spatial patterns (CBG z-score = 54.4, *p* < 0.001; watershed z-score = 5.5, *p* < 0.001). Significant hotspots of manure spills are found in the same areas of Iowa where high swine AU densities cluster (Figure 5). Hotspots of manure spills in CBGs and watersheds are observed in northwestern and north-central portions of Iowa.

**Figure 6:**
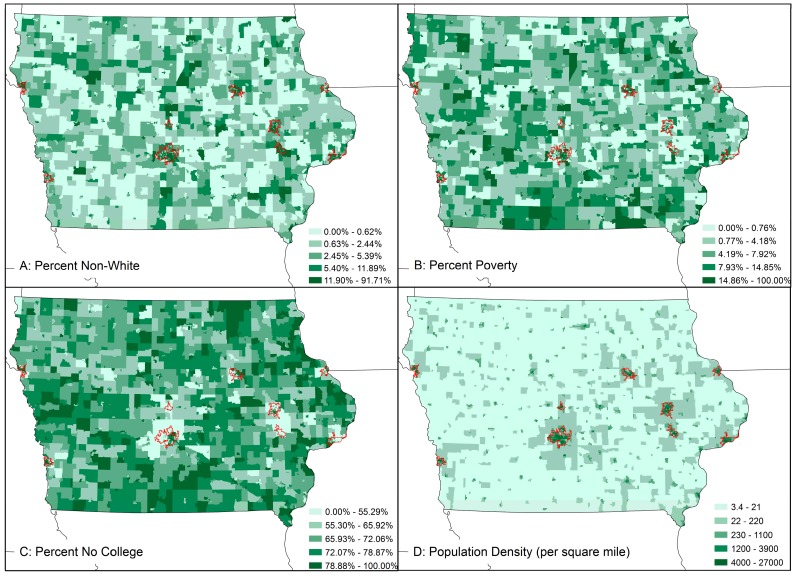
Spatial distribution of EJ covariates percent non-white residence (**A**); percent residents living in poverty (**B**); percent no college degree (**C**); and population density (**D**) in Iowa CBGs. Urban areas are outlined in red.

Descriptive statistics for Census EJ variables and swine AU densities indicate a highly varied human and swine geography in Iowa (Table 1). Swine densities in CBGs vary from zero (in urban areas) to over 2000 swine/square mile in some parts of the state. While the overall mean percentage of non-white and poor populations in Iowa CBGs are low (7.85 and 9.06, respectively, some CBGs in Iowa have no non-white or poor residents, while others have nearly all non-white and poor residents. The range for the percentage of population with less than a college education is similarly wide (9.2–100) and some parts of the state are very densely populated and some CBGs have very low population density. The spatial distributions of these variables are shown in Figure 6. Swine densities in CBGs and HUC-8 watersheds are also highly variable, as has been previously explored in hotspot analysis.

**Figure 7:**
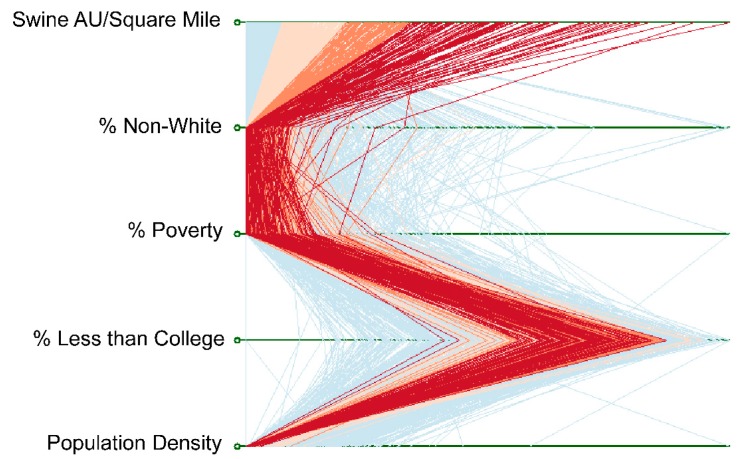
Parallel coordinate plot showing the relationship between Census block group observations of swine AU density and traditional EJ variables, as well as population density. Each line is a CBG. Red and orange lines highlight the characteristics of CBGs with the highest standard deviations of swine densities.

Parallel coordinate plotting was used to visualize the relationship between the raw data on swine AU densities in CBGs and the traditional EJ variables (non-white populations, poverty and low education) and population density in those CBGs in Iowa (Figure 7). Parallel coordinate plots are useful for visualizing patterns in data across multiple variables, in this case simultaneously visualizing for every CBG in Iowa the value of swine AU densities in an individual CBG (each line is a CBG) versus the EJ variable values for that CBG. The overall pattern in the chart suggests that those Iowa CBGs within the highest two standard deviations of the distribution of swine AU density are also CBGs where low percentages of people living in poverty or who are not white reside but where the population has a high percentage of residents with less than a college education. At the same time, the highest standard deviations of swine density are observed in CBGs with low population density.

Bivariate OLS regression indicates a highly significant relationship between swine density and EJ variables and population density (Table 2). However, diagnostics (not shown) for spatial dependence in each of the four bivariate models suggests that a spatial regression will better fit the data. When a spatial lag model is implemented, the coefficients become dramatically smaller and the significance values are also moderated while goodness-of-fit indicators improve.

Spatial multivariable regression indicates that as swine AU density in Iowa CBGs increases, the percent of residents who are non-white decreases significantly, and the percent of residents with less than a college education increases significantly (Table 3). A negative relationship is observed with the percentage of residents who live in poverty, but this relationship is not statistically significant as it was in the bivariate regression. When the population density of CBGs is included in the model (Model 2), to account for widely varying densities between rural and urban CBGs, the relationship between high swine AU density and non-white populations lost statistical significance, although the sign of the coefficient remains negative. Population density is highly significantly related to swine density: as human density increases swine density decreases. The percent of population with less than a college education retains a significantly positive relationship. In both models the spatial autoregressive coefficient (W_Swine/SqMile) is positive and statistically significant. This parameter indicates the effect of the dependent variable (swine density) in neighboring CBGs on the dependent variable in each CBG and is a parameter unique to the spatial lag model.

While EJ variables and population density could be expected to covary in such a way as to introduce multicollinearity into a model, when the three EJ variables are included (OLS version of Model 1, not shown) and population density is added (OLS version of Model 2, not shown), the multicollinearity condition number in GeoDa remains well under 30 and the VIF scores are less than ten. This indicates that the multivariable models are not subject to the problems multicollinearity generates for the reliability of the estimation of coefficients.

The results of the regression analysis indicate that traditional EJ populations, those that are poor or racial/ethnic minorities, are not exposed to high swine AU densities in Iowa as they are elsewhere in the U.S. However, the Percent No College variable was statistically significant for both models. Educational attainment variables are often included in EJ distributive analyses as an element of socioeconomic status, along with income, wealth, and occupation. At its core, socioeconomic status is a measure of access to desired resources in a socially-stratified society [67,68]. The underlying logic is that the knowledge, capabilities, and skills associated with educational attainment enable those with higher attainment levels to access greater opportunities, social positioning, and decision-making power in a market economy. The results of this analysis suggest that educational resources in Iowa, where nearly all adults complete a high school degree but many do not go on to complete a college degree, may operate independently of poverty in a manner that enables those with higher attainment levels to avoid areas of high CAFO density.

**Figure 8:**
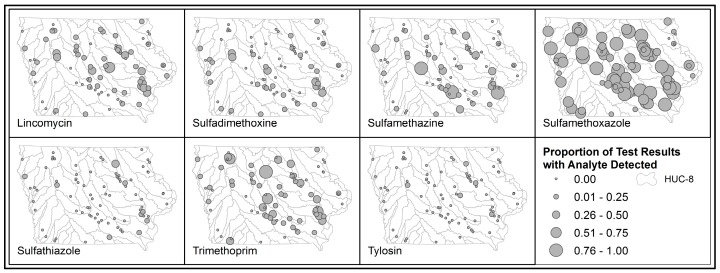
Locations of sites testing water quality in Iowa, with the proportion of samples testing positive for each of seven antibiotics.

The clustering of swine and manure spills in specific watersheds suggests that there could be downstream consequences that are more complex and difficult to capture, and that impact populations who are not in proximity to swine confinements. In thinking about environmental justice concerns associated with high levels of swine production, one potential downstream consequence of high swine AU density in Iowa is the presence of antibiotics used in swine production in public water sources. Mapping the percentage of water samples that tested positive for seven antibiotics commonly used in swine production indicates that some parts of the state more frequently have antibiotics at detectable levels in surface water (Figure 8). Sulfamethoxazole and trimethoprim in particular had high numbers of sample locations that repeatedly tested positive for those analytes. Lincomycin and sulfamethazine also had some locations in the state with many repeat detections.

**Figure 9:**
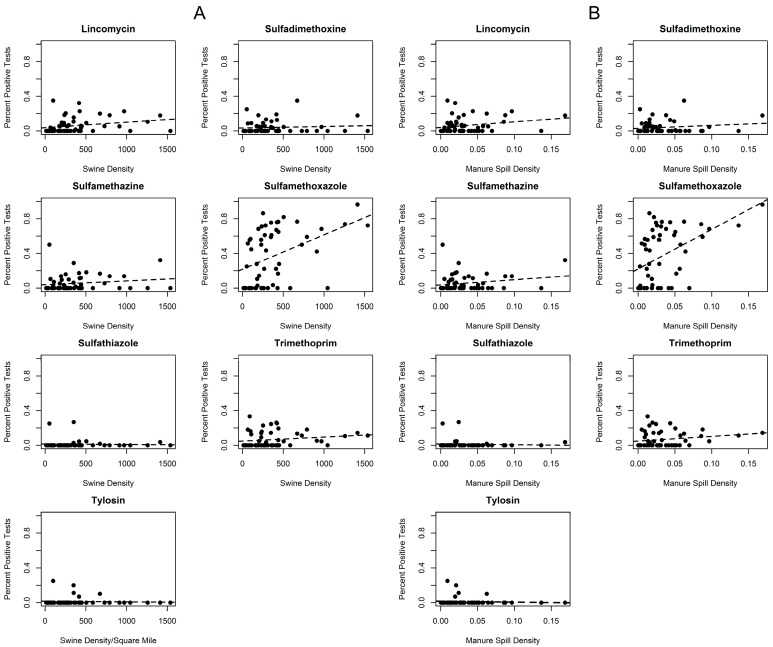
Percentage positive tests for antibiotics in watersheds versus swine AU density in watersheds (**A**) and manure spill density in watersheds (**B**). A linear regression line is fitted for each antibiotic.

Plotting the percentage of tests in a watershed that were positive for each antibiotic versus the swine AU and manure spill densities in those watersheds indicates some possible associations that need to be further explored (Figure 9). Sulfamethoxazole, lincomycin, sulfamethazine and trimethoprim in particular appear to exhibit a positive correlation between increased presence of antibiotics and higher densities of swine and manure spills.

## 4. Discussion

Swine production in CAFOs in Iowa is not evenly distributed across space, but rather clusters in certain parts of the state. Census block groups and watersheds in northwestern and southeastern Iowa are home to high densities of swine. These areas are also where there are significant hotspots of hog manure spills; the presence of manure spill hotspots indicates that there is uneven exposure to the negative impacts of uncontrolled manure release.

The unplanned and uncontrolled release of manure, which can result in fish kills in local waterways, as well as the spread of antibiotics, bacteria, viruses and noxious fumes, is one negative externality of the intensity of swine production in Iowa. Mapping the presence of antibiotics used in swine production in Iowa waterways suggests that some of the watersheds of the state that are host high densities of swine production and manure spills are also those with higher percentages of samples reporting detectable lincomycin, sulfamethoxazole and other antibiotics. Correlation does not equal causation, and these antibiotics are also used in human populations, so determining the exact source of antibiotics in waterways and their connection to upstream animal production is work that requires more attention. Additionally, the antibiotics in this analysis represent a small fraction of those used in U.S. livestock production. Not all antibiotics in use in the swine industry, such as tetracycline, are present in the data collected by the Iowa Geological and Water Survey Bureau and reported in the STORET/WQX database [69]. We are thus limited in our ability to explore the relationship between antibiotic presence and swine density.

The traditional EJ literature has linked uneven exposure to negative externalities of agriculture, traffic, and industry to higher proportions of minority and low-income populations. In Iowa, however, a different system is observed than has previously been documented with swine CAFO exposure in North Carolina. This is because the population geography of Iowa is quite different than that of North Carolina, or the south more generally. Minorities in Iowa and other parts of the Upper Midwest (Illinois, Indiana, Minnesota, etc.) are located primarily in urban, and non-CAFO appropriate, areas. Rural Iowa often has lower poverty rates than does urban Iowa, because of the high agricultural revenue associated with corn and soy production.

We find no significant relationships between swine AU density and poverty in Iowa, and a significantly negative relationship between non-white populations and swine AU density that disappears when population density within CBGs is controlled for. This suggests that a more nuanced perspective on potential EJ concerns is necessary when considering intensive livestock production in the Upper Midwest, that the differences between the rural population geography of Iowa and the Upper Midwest and that of the Southeast (North Carolina, etc.) create different “EJ populations” than are traditionally considered.

The findings align somewhat with those of Boone (2002), who in a study of Baltimore found that toxic facilities were most associated with working class white neighborhoods [70]. However, this result was attributed to historical patterns of residential segregation and discrimination in industrial employment. Similarly, Zwickl et al. (2014) found whites in the Mid-Atlantic region had higher exposure to air toxics, although this was attributable to differing distributions in race/ethnicity across the cities of the region [71]. For CAFOs in Iowa, power differentials endemic to rural agricultural settings offer a more likely potential explanation. Rural residents who themselves are not engaged in agricultural production, or who favor retaining an older style of independent farming rather than corporate farming, express fears of air and water pollution, negative health impacts and reduced property values [72,73]. It should be noted that these concerns are not limited to Iowa or the U.S., the tension between local communities and CAFOs plays out in many sites of intensive, globalized and integrated animal production, such as Canada and Europe [74,75,76,77,78,79,80,81].

Despite concerns and fears that are articulated in similar ways across a diversity of rural settings, rural communities may lack regulatory, social, and organizational resources to restrict exposure to environmental hazards associated with industrialized agriculture, due to the dominance of commodity organizations in rural policy making [48,49,82]. Recent decisions by the Iowa Supreme Court limit local capacity to regulate or otherwise limit CAFO operations, and the Iowa General Assembly has attempted to create a legal environment that is protective of industrial animal production [12,72]. Furthermore, the political economy of industrialized animal production may mean there are limited other economic development options for rural agricultural communities to pursue, a dynamic that has been found with other environmental hazards [15]. Collectively, these factors support a framing for EJ in rural settings in which the primary axes of decision-making power operate independent of race, ethnicity, and income.

The presence of clustering of swine production and manure spills within watersheds, and in particular watersheds that serve major cities in Iowa, such as Des Moines and Sioux City (Figure 4B and Figure 5B), indicate that populations downstream from swine CAFOs are at risk of exposure to externalities from high densities of swine upstream. Swine CAFOs do not represent the only threat to water quality in downstream cities, as evidenced by the ongoing lawsuit by the city of Des Moines against upstream corn and soy producers whose agricultural pollution has contributed to poor water quality for city residents [83]. It should also be noted that Iowa is a leading site of egg production, 50 million layer hens were in the state in 2015, and is also home to approximately four million cattle used in beef and dairy production [5]. The U.S. government is currently suing an Iowa cattle farmer for Clean Water Act violations, namely the illegal discharge of manure [84]. Iowa’s waterways are susceptible to pollution from multiple animal and agribusiness sources and Iowa’s rural populations are exposed to the externalities of multiple types of animal operations.

### Data Needs for EJ CAFO Research

Understanding downstream or long-term EJ consequences of high swine concentrations relies on high quality and publicly available datasets. The importance of quality data and information, as well as the involvement of the public in planning processes, are highlighted as critically important in the ability of rural communities to engage fairly and fully with the siting of CAFOs [74,79,85]. Comprehensive reporting of not only the locations of swine confinements and the number of swine in them, but also the dates of establishment and closing of the confinement, the location of waste disposal sites (i.e., what fields are being sprayed with the manure from that confinement, how often and in what amount) and the elevation of the confinement are needed. The Iowa DNR database on CAFOs is better than some; Illinois, for instance, has no publicly available data on CAFO locations, despite raising 7% of U.S. hogs. The Iowa DNR site does not, however, have the date of construction or implementation for many (most) of the swine CAFO records. The reliability of the manure spill data is also problematic; for instance, one day in January 2009 reports 234 swine manure spills. A 2012 summary of data availability on CAFOs, produced by the EPA, shows vastly different availability of data across U.S. states [86]. Inconsistencies in reporting on CAFO locations, herd sizes, dates of operation and other factors makes comparison of CAFO patterns between states difficult, if not impossible.

The lack of publicly available data on the demographics of land tenure and farm operators at scales smaller than counties limits understanding of who is subject to “downstream” impacts. Based on the results of our analysis, does the distribution of socio-demographic characteristics associated with higher CAFO density reflect wealthier, but less educated, white farm owners? The answer to this question is unclear. Linking socio-demographic characteristics with land tenure can be complicated in rural Iowa, given substantial numbers of farmers employed by agri-business and farmers who sow land rented from other landowners. Land value is an important physical asset that is not captured by financial indicators of poverty and income, but could also be important for understanding access to resources in the context of EJ. Improved spatially explicit data on the demographics of land tenure would enable construction of a wealth indicator. Wealth combines physical and financial assets, and its relationship with income may vary with age and minority status [87].

Swine CAFOs located within the 100 year plus 1 foot floodplain are required to be elevated so that they do not experience flooding and release of manure during floods, but the elevation of CAFOs is not included in the database. Digital flood plain maps approved by the U.S. Federal Emergency Management Agency (FEMA) are also unavailable for many Iowa counties that contained hotspot CBGs of high swine density, so analysis of the potential risk of manure release due to flooding is not possible. Documenting the potential for unevenly distributed negative impacts on drinking water is also difficult, given that there is no comprehensive dataset for private wells in Iowa. It is likely that the rural areas of Iowa that are hotspots for swine production also contain a high percentage of residents who drink untreated groundwater from private wells. Assessing a relationship is impossible, however, with currently available data. Even the manure spill data used in this analysis would pose greater risk to surface than groundwater sources.

The data limitations present in our analysis only serve to highlight the broader limitations in assessing EJ concerns in swine production in Iowa. Iowa is a state with a government that is very friendly to swine production, as well as very friendly to corn and soy production. Corn and soy production have similar, albeit different, potential for negative impacts on water quality in Iowa’s ground and surface waters.

## 5. Conclusions

The concentration of swine into larger farms in smaller amounts of land results in potential concentration of the negative external consequences of intensive livestock production. Such negative externalities include not only air and water pollution but also potential for negative or unevenly shared social and economic repercussions. In Iowa, swine production and manure spill densities vary widely across both CBGs and watersheds. While traditional EJ populations of non-whites and poor residents are not associated with high swine density in Iowa as they are in other parts of the U.S., the importance of education in providing a buffer against proximity to swine is observed. The population landscape of Iowa and other top Midwestern swine producing states necessitates a more complex appraisal of the potential for environmental injustice or inequity in bearing the burden of swine production, in examining surface water contamination for example. Upstream policies that ease and streamline CAFO establishment and operation have downstream consequences on proximal and distal populations exposed to the risks of swine production but not necessarily its benefits. More broadly speaking, the people of Iowa perhaps bear the burden of the environmental injustice of growing pork and corn and soy not only for the U.S. but also the world.

## Figures and Tables

**Table 1:** Descriptive statistics for swine density and EJ variables in Census Block Groups and watersheds.

Variable	Mean	Minimum	Maximum	Std. Deviation	*N*
Swine AU/Square Mile (CBG)	202.45	0.00	2725.84	363.30	2630
Swine AU/Square Mile (HUC-8)	371.79	12.72	1537.18	339.01	56
Percent Non-White (CBG)	7.85	0.00	91.70	11.35	
Percent Poverty (CBG)	9.06	0.00	100.00	10.38	
Percent No College (CBG)	66.37	0.00	100.00	15.72	
Population Density/Square Mile (CBG)	1845.50	3.44	26899.40	2624.55	

**Table 2:** Results of the OLS and spatial lag bivariate regressions spatial multivariable regressions indicating the relationship between swine AU density and EJ variables and population density in CBGs without considering the influence of other covariates. AIC = Akaike Information Criterion (smaller = better fit); LL = log likelihood (larger = better fit).

Variable	OLS				Spatial Lag			
	Coefficient	*p*-Value	AIC	LL	Coefficient	*p*-Value	AIC	LL
Percent Non-White	−652.18	<0.001	29,380.3	−14,688.2	−160.26	0.002	27,605.1	−13,799.5
Percent Poverty	−430.36	<0.001	29,413.2	−14,704.6	−115.37	0.027	27,609.9	−13,802.0
Percent No College	361.33	<0.001	29,391.1	−14,693.5	74.37	0.018	27,609.2	−13,801.6
Population Density	−0.055	<0.001	29,292.3	−14,644.1	−0.013	<0.001	27,594.1	−13,794.0

**Table 3:** Results of the spatial multivariable regressions indicating the relationship between EJ variables and swine AU density in CBGs (Model 1) and the relationship between EJ variables and swine AU density after controlling for population density (Model 2). Statistically significant variables are bolded. AIC = Akaike Information Criterion; LL = log likelihood.

**Model 1**				
**Variable**	**Coefficient**	**Std. Error**	**z-Value**	***p*-Value**
W_Swine/SqMile	0.786	0.014	52.993	0.000
Constant	−23.132	21.751	−1.063	0.287
**Percent Non-White**	**−121.147**	**56.102**	**−2.159**	**0.031**
**Percent No College**	**95.765**	**32.958**	**2.906**	**0.003**
Percent Poverty	−112.504	59.524	−1.890	0.058
AIC = 27,599.6, LL = −13,794.8				
**Model 2**				
**Variable**	**Coefficient**	**Std. Error**	**z-Value**	***p*-Value**
W_Swine/SqMile	0.779	0.015	51.620	0.000
Constant	−3.467	22.639	−0.153	0.878
Percent Non-White	−67.665	58.853	−1.149	0.250
**Percent No College**	**73.952**	**33.857**	**2.184**	**0.029**
Percent Poverty	−87.767	60.136	−1.459	0.144
**Population density**	**−0.009**	**0.003**	**−3.106**	**0.002**
AIC = 27,592.2, LL = −13,790.1				

## Supplementary Materials

The following are available online at www.mdpi.com/1660-4601/13/9/849/s1, Figure S1: Education indicators in Iowa CBGs.
