# Supplementary material for: Pigs in Space: Determining the Environmental Justice Landscape of Swine Concentrated Animal Feeding Operations (CAFOs) in Iowa
Source: Int J Environ Res Public Health. 2016 Aug 25;13(9):849. doi: 10.3390/ijerph13090849 (PMC5036682; doi:10.3390/ijerph13090849)
Supplement: Supplementary file 1 [file ijerph-13-00849-s001.pdf]

Supplementary Materials: Pigs in Space: Determining the Environmental Justice Landscape of Swine Concentrated Animal Feeding Operations (CAFOs) in Iowa

Margaret Carrel, Sean G. Young and Eric Tate

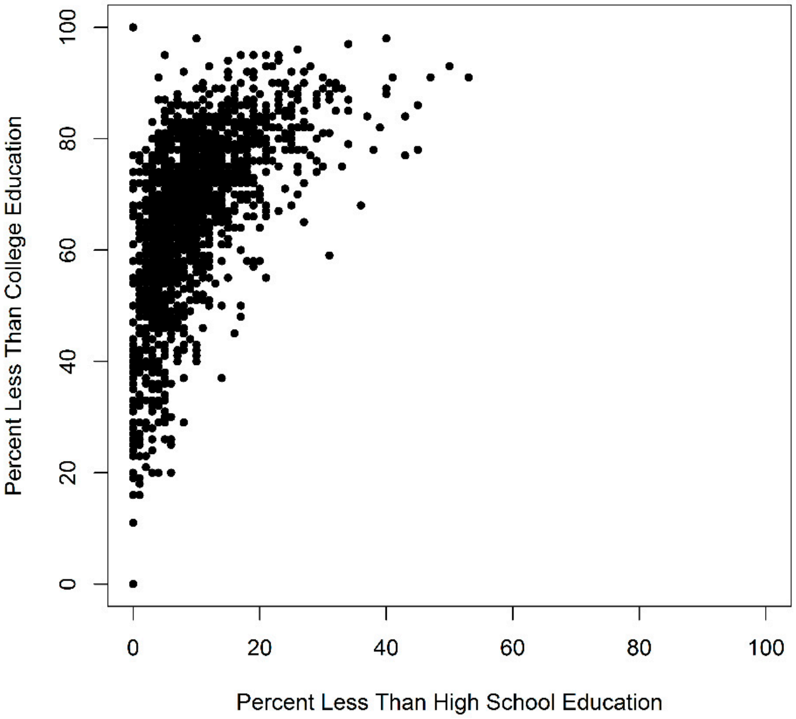

Figure S1. Education indicators in Iowa CBGs.

© 2016 by the authors; licensee MDPI, Basel, Switzerland. This article is an open access article distributed under the terms and conditions of the Creative Commons by Attribution (CC-BY) license (http://creativecommons.org/licenses/by/4.0/).
